# COMPARATIVE EVALUATION OF FIRST- AND ADAPTED SECOND-GENERATION BONE CEMENTATION IN POST-CURETTAGE BONE DEFECT FILLING IN AN ANIMAL MODEL

**DOI:** 10.1590/1413-785220263405e301195

**Published:** 2026-08-10

**Authors:** Mateus de Vita Mendes Cruz, Nelson Fabrício Gava, Vitor Freire da Rocha, Cátia Isumi Miyase, Edgard Eduard Engel, Mariana Avelino dos Santos

**Affiliations:** 1 Universidade de Sao Paulo, Faculdade de Medicina de Ribeirao Preto (HCFMRP), Hospital das Clinicas, Departamento de Ortopedia e Anestesiologia, Ribeirao Preto, SP, Brazil.; 2 Centro Universitario Padre Albino, Hospital Escola Emilio Carlos e Hospital Padre Albino, Departamento de Ortopedia do Catanduva, Catanduva, SP, Brazil.; 3 Universidade de Sao Paulo, Faculdade de Medicina de Ribeirao Preto (HCFMRP), Hospital das Clinicas, Ribeirao Preto, SP, Brazil.

**Keywords:** Bone Cements, Curettage, Polymethyl Methacrylate, Bone Neoplasms, Cimentos Ósseos, Curetagem, Polimetil Metacrilato, Neoplasias Ósseas

## Abstract

**Introduction:**

Bone cementation is fundamental for orthopedic stability, and different techniques have been proposed to optimize the bone–cement interface.

**Objective:**

To evaluate whether adapted second-generation cementation improves osteointegration compared with the first-generation technique.

**Methods:**

Bone defects created in the femora and tibiae of goats were filled with polymethylmethacrylate using either the first-generation technique (Group 1) or an adaptation of the second-generation technique (Group 2). Cementation quality and the bone–cement interface were assessed by radiography, CT, and photographic analysis, evaluated by orthopedic examiners. Statistical analyses employed the chi-square and Mann–Whitney U tests.

**Results:**

No statistical differences were observed between the groups regarding cementation quality (radiography and CT) or osteointegration (photographic analysis) (p > 0.05). On CT, the mean number of bubbles was higher in the second-generation (6.05 vs. 3.95), whereas the proportion of samples with ≥5 bubbles was higher in the first-generation (50% vs. 30%). Photographic analysis showed that the second-generation group had a higher mean number of interdigitations (2.4 vs. 1.6) and a greater frequency of rough interfaces (50% vs. 25%). However, the first-generation group presented greater interdigitation depth (0.40 mm vs. 0.35 mm).

**Conclusion:**

Adapted second-generation cementation showed no clear advantage over the first-generation technique. ***Level of evidence II; Investigation of treatment outcomes in an animal model.***

## RESUMO

**Introdução:**

A cimentação óssea é fundamental para a estabilidade ortopédica, e diferentes técnicas têm sido propostas para otimizar a interface osso-cimento.

**Objetivo:**

Avaliar se a cimentação de segunda geração adaptada promove melhor osteointegração que a técnica de primeira geração.

**Métodos:**

Falhas ósseas produzidas em fêmures e tíbias de caprinos foram preenchidas com polimetilmetacrilato por técnica de primeira geração (Grupo 1) ou por adaptação da segunda geração (Grupo 2). A interface osso-cimento foi analisada por radiografia, TC e fotografias por ortopedistas. As análises estatísticas empregaram os testes qui-quadrado e Mann-Whitney U.

**Resultados:**

Não houve diferenças estatísticas entre os grupos quanto à qualidade da cimentação (RX e TC) ou à osteointegração (análise fotográfica) (p > 0,05). Na TC, a média de bolhas foi maior na segunda geração (6,05 vs. 3,95), mas a proporção de amostras com ≥5 bolhas foi superior na primeira (50% vs. 30%). Nas fotografias, a segunda geração apresentou maior número médio de interdigitações (2,4 vs. 1,6) e maior frequência de interfaces rugosas (50% vs. 25%); no entanto, a profundidade média das saliências foi maior no grupo da primeira geração (0,40 mm vs. 0,35 mm).

**Conclusão:**

A cimentação de segunda geração adaptada não demonstrou vantagens sobre a de primeira geração. ***Nível de evidência II; Investigação dos resultados de tratamento em modelo animal.***

## INTRODUCTION

Benign bone tumors exhibit variable histological aspects and biological behaviors. Although rarely lethal, they can result in significant changes to bone architecture with potential mechanical weakening^1,2^. Among the various therapeutic possibilities, curettage, associated with adjuvant techniques that extend the necrosis of neoplastic cells beyond the curetted margins, is the most frequent treatment option, as it reduces the local recurrence rate and offers good functional results^3–7^. Orthopedic cement or polymethylmethacrylate (PMMA) is used both for filling and mechanically stabilizing bone defects created by curettage, as well as for its adjuvant action^4–7^.

Cementation in orthopedics was introduced by Sir John Charnley in 1958 for the fixation of the femoral and acetabular components in total hip arthroplasty^8–10^. The first-generation cementation technique, known as "*finger packing"*, involved the manual mixing of the cement and its insertion into the medullary canal by finger pressure^8^. During the 1980s, the technique was refined to provide greater pressure during its insertion. Low-viscosity cement began to be introduced retrogradely, after careful cleaning and occlusion of the medullary canal, with the aid of a syringe and gun^9,11^. These adaptations and improvements defined the second-generation cementation, reducing the presence of blood at the bone-cement interface and improving the penetration of the cement into the bone pores^8^. Pressurization during application resulted in greater integration of the materials and better mechanical stability, with lower rates of loosening of the prosthetic components and a reduced need for surgical revisions^12^.

In the field of orthopedic oncology, the use of PMMA for filling cavities after curettage was first described in 1969, in the treatment of giant cell tumors (GCT)^13,14^. With successful application, the technique has been expanded to treat other benign tumors^15^. In addition to being an accessible and low-cost material, with a simple application technique for filling irregular bone defects, cementation provides mechanical stability, and in subchondral lesions, it preserves joint mobility^16,17^. The cement completely fills the cavity without leaving dead space, offers immediate support, preventing collapse, and also penetrates the bone, forming interdigitation that provides better coupling at the bone-cement interface^14,18,19^. The adjuvant action of PMMA results from polymerization, which is an exothermic reaction and causes tissue necrosis up to 2–3 mm deep, providing better control of recurrences^18,20,21^.

Despite advances in cementation techniques, the first generation is still universally used for filling irregular cavities after curettage. However, loosening of the cement block is a frequent complication, resulting in the formation of a fibrous tissue membrane between the bone and the cement, radiographically characterized by a radiolucent halo^22–25^. Several causes are related to this loosening, such as cementation technique, preparation of the receiving bed, thermal necrosis, and mechanical incompatibility^26^. Therefore, it is possible that the application of cement in a lower viscosity state and under high pressure, with benefits already well established in the context of arthroplasties, may replicate good results in oncology^9, 27^.

The objective of this study is to evaluate, in an animal model, whether adapting the second-generation technique for post-curettage defects can improve the initial quality of cementation, increasing integration at the bone-cement interface.

## MATERIALS AND METHODS

In this experimental animal study, with approval from the Animal Ethics Committee (CEAU) of the *Campus* of Ribeirão Preto-USP (Protocol No. 06.1.254.53.0), defects were created in the femurs and tibias of sheep to simulate the curettage of bone tumors. These defects were then filled with bone cement, using the first-generation technique in group 1 and an adapted second-generation technique in group 2. The quality of cementation was evaluated based on parameters such as interdigitation between the cement and the bone, the presence of spaces between both, and the amount of air bubbles in the cement, analyzed through radiographs, computed tomography, and photographs of the specimens.

Each group consisted of 10 young sheep of the same sex of the Santa Inês breed^22^ that, after intravenous anesthesia with ketamine and xylazine, asepsis, and lateral incision of the knee, underwent the creation of cubic bone defects of 15 mm on each side (3.375 cm³) in the metaepiphyseal regions of the femur and tibia, followed by abundant washing with 0.9% saline solution and 0.1% heparin until the macroscopic elimination of clots.

In group 1, the cavities were filled using the first-generation cementation technique, with the application of orthopedic cement (PMMA) (C-MAXXX®, Cimtech®, Rio Claro, Brazil) in a higher viscosity state – after the setting time – inserted manually and compacted with fingers. In group 2, an adaptation of the second-generation cementation was used. PMMA was applied in a lower viscosity state - before the setting time, still in the liquid phase – followed by pressurization using two surgical gloves (one inside the other) filled with water that occluded the bone window and transmitted the pressure applied in the glove to the cement. In this way, an attempt was made to occlude the bone window while simultaneously increasing the pressure inside the cavity, pushing the cement into the pores of the bone. The pressure of the glove against the opening was maintained until the cement fully cured.

**Figure 1:**
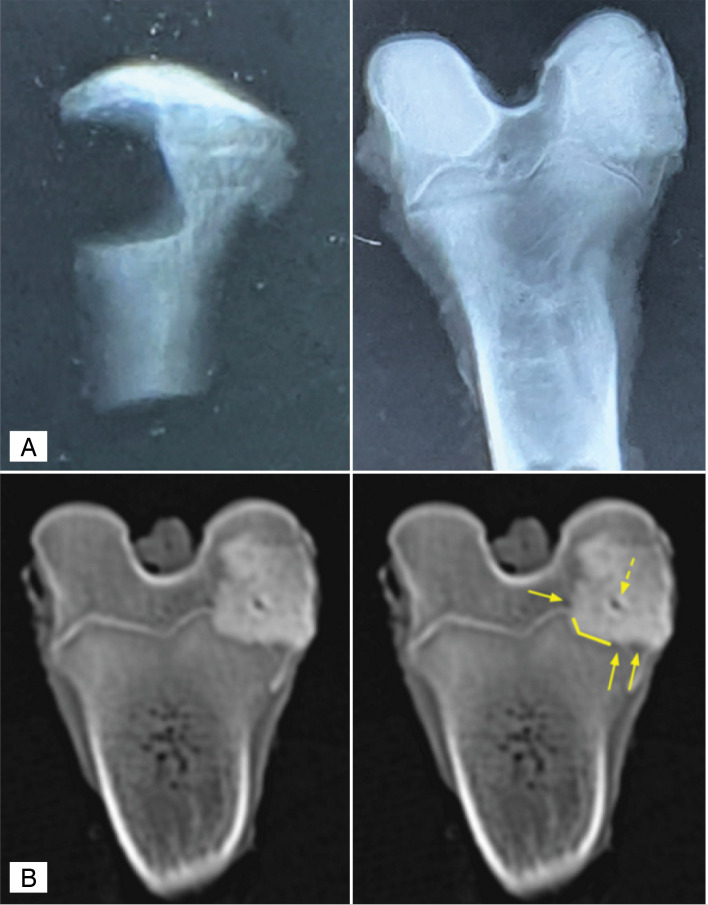
Bone defects in X-ray and computed tomography.

After the procedure, the animals were euthanized, and the bone segments containing the cemented cavities were resected. These segments underwent simple radiographic examination (X-ray) and computed tomography (CT) (Figure 1A). Subsequently, the segments were sectioned in the coronal plane, cleaned, and photographed with a high-resolution digital camera.

A. Simple X-ray of a bone defect in the metaphyseal region of the tibia, and of a bone defect filled with PMMA in the epiphyseal region of the femur. B. Bone defect in CT: the solid arrow indicates the space between cement and bone (lacuna), the solid line shows the interface between the materials (transition), and the dashed arrow indicates the presence of bubbles.

**Figure 2:**
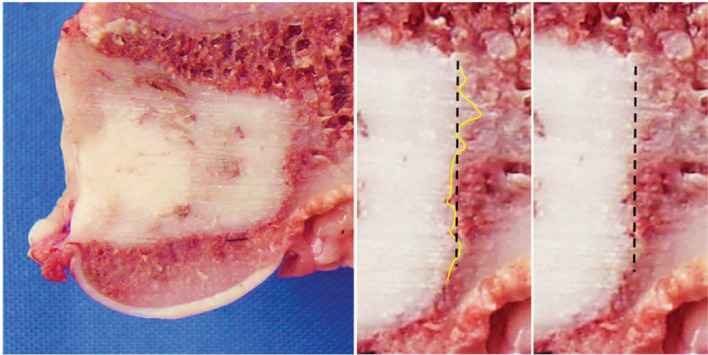
Analysis of the interdigitations.

The quality of the bone-cement interface was evaluated based on two parameters in the radiographs and tomographies: the presence of space between the bone and the cement, referred to as a "lacuna," and the appearance of the interface between the materials, called "transition." The transition was classified as abrupt, with a clear distinction between the bone and the cement, or smooth, indicating better interdigitation of the cement into the bone (Figure 1B). Additionally, the presence and appearance of the interdigitation (penetration of the cement into the pores of the trabecular bone) were analyzed in photographs. To standardize the evaluation, the central 10 mm of the deep face of the cubic bone defect was examined. With the aid of the ImageJ program (version 1.49, U.S. National Institutes of Health, Bethesda, Maryland, USA), a mean line was drawn along the perimeter of the cement and another line parallel to the initial one, outlining the interdigitation perpendicular to the initial line. Each deviation from the mean line was considered a prominence. The number of prominences and their size in mm were quantified (Figure 2). The penetration of the interdigitation in the piece was also considered as "deep" (average size of the prominences ≥ 0.5 mm) or "superficial." Finally, a parameter was created for the classification of the interface in the CT and in the photograph, as "rough" (many prominences, ≥ 3) or "smooth." To assess the quality of the cement production, the number of bubbles present in the cement block was measured.

Photographic analysis in the coronal plane of the samples. In the linear dotted line, the central surface of 10 mm of the bone defect; and in the yellow line, the assessment of the interdigitations (protrusions).

All images were analyzed blindly and independently by two to three experienced orthopedic examiners.

For data evaluation, Microsoft Excel Corporation software (2023) and Jamovi (version 1.6) were used. Qualitative variables were analyzed using the chi-square test, and quantitative variables were analyzed using the Mann-Whitney U test, considering that they presented a non-parametric distribution. The OpenAI tool, ChatGPT version GPT-4, was used exclusively for language corrections during the writing process of the article.

## RESULTS

### Simple Radiography

The radiographic analysis did not reveal a statistical difference between the groups for the evaluated parameters (p > 0.05). The gaps were identified in 75% of the first-generation samples and in 90% of the second-generation. The transition was considered abrupt in 50% and in 30% of the cases, respectively. In all samples of group 2, bubbles were identified (100%), while in the other group, in 90% of the cases (Table 1).

**Table 1:** Lacuna, transition, and bubbles in simple X-rays according to the type of cementation.

Parameter	1st generation	2nd generation	N(t)	N(a)	p
Gaps (Yes, %)	75	90	10	60	0.15*
Transition (Abrupt, %)	50	30	10	40	0.19*
Bubbles (Yes, %)	90	100	10	40	0.14*

N(t), total number of samples analyzed in each group. N(a), total number of evaluations conducted by different examiners.

* chi-square.

### Computed Tomography

The gaps were present in 45% of the first-generation samples and 55% of the second (p > 0.05). Regarding the transition at the bone-cement interface, 23.33% of the first-generation samples were rough, compared with 55% of the second generation (p = 0.014). The average number of bubbles was higher in group 2 (6.05) than in group 1 (3.95). When grouped into many (≥ 5) and few bubbles, 50% of the samples in group 1 had many bubbles, in contrast to 30% in group 2. For these two analyzed parameters, there was no statistical difference (p > 0.05) (Table 2).

**Table 2:** Gap, transition, and bubbles in computed tomography according to the type of cementation.

Parameter	1st generation	2nd generation	N(t)	N(a)	p
Gaps (Yes, %)	45	55	10	40	0.52*
Transition (Rough, %)	23.33	55	10	60	0.014*
Bubbles (average, number)	3.95	6.05	10	40	0.17**
Bubbles (many, %)	50	30	10	40	0.33*

N(t), total number of samples analyzed in each group. N(a), total number of evaluations conducted by different examiners.

* chi-square.

** Mann-Whitney U.

### Photographs

The average number of protrusions per sample in group 1 was 1.6, while in group 2 it was 2.4. As with the other parameters, this difference was not significant (p > 0.05). The average size of the protrusions was 0.40 mm and 0.35 mm, respectively in groups 1 and 2. In the first generation, 25% of the samples showed many protrusions and 50% in the second generation. The penetration was deep in 30% of the samples in group 1 and 15% in group 2 (Table 3).

**Table 3:** Analysis of protrusions in photographic images according to the type of cementation.

Parameter	1st generation	2nd generation	N(t)	N(a)	p
Average (number)	1.6	2.4	10	40	0.07**
Average (mm)	0.40	0.35	10	40	0.69**
Quantity (many, %)	25	50	10	40	0.10*
Penetration (deep, %)	30	15	10	40	0.25*

N(t), total number of samples analyzed in each group. N(a), total number of evaluations conducted by different examiners.

* chi-square.

** Mann-Whitney U.

## DISCUSSION

The results suggest that the adapted second-generation cementation technique does not present significant advantages compared to the first-generation technique. Despite the trend towards more protrusions/interdigitation in the second-generation cementation group, more gaps were also found in this group, indicating that the adaptation of the second-generation technique did not provide more efficient interdigitation of the cement in the bone cavity, although there were no significant differences in these parameters. Regarding the quality of the cement, analyzed from the quantity and size of the bubbles, there was no difference between the techniques; therefore, no interference in the strength or adhesion of the cementation.

The second-generation cementation presupposes the creation of a closed compartment in which low-viscosity cement is injected in a liquid state under high pressure. In the medullary canal for implanting prostheses, these conditions are achieved by obstructing the canal with a plastic pin (*stop*), and the opening of the medullary canal is blocked with a plastic and *rubbery* device that surrounds the syringe tube^28^. In the defects created after curettage of bone tumors, the opening is a cortical window that is usually large and of variable shape, making it difficult to develop specific equipment. The double-glove filled with water should occlude the cortical window and transmit the applied pressure to the cement, pushing it into the pores of the trabecular bone. The study data show that this did not occur, and the likely cause is that the pressure applied to the glove was neither sufficient nor comparable to the pressure achieved with the use of a gun. It is possible that developing a technique that allows greater pressure to be applied to the cement will improve the quality of integration. This is, therefore, an open field for development.

The adoption of more modern cementation techniques for treating cavities after curettage remains a challenge. Developing mechanisms that increase pressure in irregular cavities requires technical sophistication to adapt to different bone windows, which may increase procedural cost and cause additional damage to the bone structure if the cavity must be molded beyond the limits of curettage. In light of this, the results obtained indicate that maintaining digitopressure (first generation) is an efficient method for the proposed end, and is accessible, technically easier, and lower in cost.

## CONCLUSION

In an animal model, a comparison of first-generation cementation methods and a second-generation adaptation in bone defects simulating curettage of bone tumors showed no differences, leading to the conclusion that the first-generation technique (digitopressure), with the introduction of cement after the setting phase using fingers, remains the method of choice.

## Data Availability

The underlying contents of the research text are contained in the manuscript.
